# An examination into the safety and efficacy of Khapregesic®, a Khaya senegalensis preparation, on physical and psychological wellbeing in women experiencing menopausal symptoms: a randomised, double-blind, placebo-controlled trial

**DOI:** 10.3389/frph.2026.1824321

**Published:** 2026-06-02

**Authors:** Adrian L. Lopresti, Stephen J. Smith, Frederick R. Ferdinands

**Affiliations:** 1 Clinical Research Australia, Perth, WA, Australia; 2 College of Science, Health, Engineering and Education, Murdoch University, Perth, WA, Australia; 3 Bioactive Natural Health Pty Ltd., Perth, WA, Australia

**Keywords:** clinical trial, fatigue, herbal, hot flushes, Khaya senegalensis, menopause

## Abstract

**Background:**

Khaya senegalensis is a tree species that has traditional use for the alleviation of menstrual and hormonal symptoms in women. Moreover, supplementation with a Khaya senegalensis preparation (Khapregesic®) was associated with significant and rapid improvements in menstrual pain, mood, sleep, and fatigue in women with menstrual distress over 28 days. The present study aimed to assess the safety and efficacy of Khapregesic® over the same intervention period in women reporting menopausal symptoms across the transition from perimenopause to post-menopause.

**Methods:**

In this 28-day, randomised, double-blind, placebo-controlled study, 140 peri- and postmenopausal women aged 42–62 years experiencing menopausal-related symptoms were supplemented with 2 grams daily of Khapregesic® or a matching placebo. Outcomes were assessed using validated self-report measures of menopausal symptoms (primary outcome: Greene Climacteric Scale), sleep disturbance/sleep-related impairment (PROMIS—Sleep Scale), fatigue (FACIT-Fatigue), vasomotor symptoms (hot flush rating scale), and digestive symptoms, alongside tolerability and safety monitoring.

**Results:**

There were no statistically significant group differences in change scores across the primary and secondary outcomes. However, stratified analyses by menopausal stage indicated in postmenopausal women (*n* = 69), Khapregesic® was associated with significantly greater improvements versus placebo in psychological symptoms (*p* = 0.014), fatigue (*p* = 0.045), and sleep disturbances (*p* = 0.024). In contrast, no group differences were detected in perimenopausal women (*n* = 71). Khapregesic® for 28 days was generally well tolerated, with a tendency towards transient, mild gastrointestinal symptoms.

**Conclusions:**

Based on data from the full cohort, no statistically significant group effects were observed in this study. Subgroup analyses revealed positive improvements in post-menopausal but not perimenopausal women in mood/psychological symptoms, sleep disturbance, and fatigue. However, these secondary analyses should be considered tentative and require confirmation in future trials.

**Clinical Trial Registration**: https://anzctr.org.au/ACTRN12625000247471p.aspx, identifier ACTRN12625000247471p.

## 1 Introduction

Natural menopause is the result of a loss of ovarian follicular activity and typically begins with changes in the patterns of a woman's menstrual cycle. When it is not the result of another pathological or physiological cause, the menopausal transition typically occurs between the ages of 45 and 56 (1, 2). According to the Stages of Reproductive Aging Workshop (STRAW+10), perimenopause begins when there are persistent changes in cycle length of 7 or more days between consecutive cycles and continues for 12 months after the last menstrual period. The post-menopause phase begins immediately after 12 months of amenorrhea (3).

It is estimated that during the menopausal transition, up to 85% of women experience menopausal symptoms; however, the frequency and severity of symptoms can vary widely (4). During this transition, approximately 50%–75% of women have hot flushes, night sweats, or both, with these vasomotor symptoms lasting on average for 1–6 years, but can last up to 15 years in 10%–15% of postmenopausal women (5). Approximately 50%–75% of women experience genitourinary syndrome of menopause, comprising vaginal dryness, discomfort during intercourse (dyspareunia), urinary urgency, and other genital and urinary tract issues (4). Psychological and cognitive symptoms are also common, with up to 70% of women experiencing symptoms such as mood changes, brain fog, forgetfulness, difficulty concentrating, irritability, and anxiety (6). Other commonly experienced symptoms include fatigue, sleep disturbances, loss of libido, headaches, migraines, stomach bloating, and weight gain (4, 7). Digestive symptoms are particularly problematic in menopause, characterised by an increased severity of gastrointestinal symptoms that are believed to be due to the effect of sex hormone changes on brain-gut interactions, which, in turn, affect visceral perception and gastrointestinal function (8). In a study on women in primary care practice, altered bowel function was reported by 14% of perimenopausal women compared to 38% of post-menopausal women (9).

The management of troublesome menopausal symptoms includes non-pharmacological, nonhormonal and hormonal treatments (7, 10). In recently published clinical practice guidelines from the European Society of Endocrinology, hormone therapy was recommended, particularly for the treatment of vasomotor symptoms, although a holistic approach to treatment was encouraged (11). This included a focus on a healthy diet, regular exercise, adequate sleep, stress management, tobacco avoidance, and moderation of alcohol intake. Moreover, menopausal hormonal therapy was not recommended in women with uncontrolled hypertension, poorly controlled diabetes, or in women who have a history of hormone-dependent cancers, such as breast cancer. In a review of herbal ingredients for the treatment of menopausal symptoms, positive evidence, albeit inconsistent, has been demonstrated for plants such as Sage (*Salvia officinalis*), Lemon balm (*Melissa officinalis*), Valeriana officinalis, Black cohosh (*Cimicifuga racemosa*), Fenugreek (*Trigonella foenum-graecum*), Black cumin (*Nigella sativa*), Vitex (*Vitex agnus-castus*), and Fennel (*Foeniculum vulgare*) (12).

Khaya senegalensis (KS) is a tree species native to Africa (and now also cultivated in Australia) that has traditionally been used as an antimicrobial, anthelmintic agent and an anti-parasitic used to treat malaria, in addition to headaches, fever, rheumatism, jaundice, and epilepsy (13–16). In traditional medicine, KS has also been used to treat menstrual pain, dysmenorrhoea, ovulation disturbances, and digestive pain and discomfort. Khapregesic® is a pure KS dry stem bark that undergoes a patented, tightly-controlled process from harvest through to fine micronisation, designed to preserve a high level of active constituents while minimising contaminants. Khapregesic® is listed with the Australian Therapeutic Goods Administration (TGA) and has indications for alleviating symptoms associated with menstrual pain and irregularities, digestive disturbances, and fatigue. Although there have been no randomised controlled trials in humans on Khapregesic® for the treatment of menopausal symptoms, an unpublished observational trial of 45 women and anecdotal reports have been positive. Moreover, in a recently published clinical trial, positive therapeutic findings were identified for menopausal-like symptoms (17). In this randomised, double-blind, placebo-controlled study on 84 women aged 18 to 50 years experiencing menstrual distress, significant improvements in menstrual-related pain, psychological symptoms, sleep disturbances, and fatigue were identified. Although the recruited population had a low prevalence of vasomotor symptoms, those who did experience them reported fewer hot flushes and night sweats. KS has multiple mechanisms of action that may be beneficial for alleviating menopausal symptoms, including anti-inflammatory and antioxidant effects, and an influence on neurotransmitter activity (13, 14, 16, 18–20).

Therefore, based on the positive findings from the recently completed clinical trial, traditional evidence, positive observational evidence, and its potential bioactivity, the aim of this study was to investigate the effects of 4 weeks of supplementation with Khapregesic® in perimenopausal and post-menopausal women aged 42–62 years experiencing menopausal symptoms. It was hypothesised that supplementation would be associated with improvements in physical and psychological symptoms associated with the menopausal transition.

## 2 Materials and methods

### 2.1 Study design and procedures

This study was registered prospectively with the Australian and New Zealand Clinical Trials Registry (ACTRN12625000247471p). Ethics approval was obtained from the National Institute of Integrative Medicine Human Research Ethics Committee (approval number 0154E_2025), and informed consent was obtained from all participants before beginning the study.

**Figure 1:**
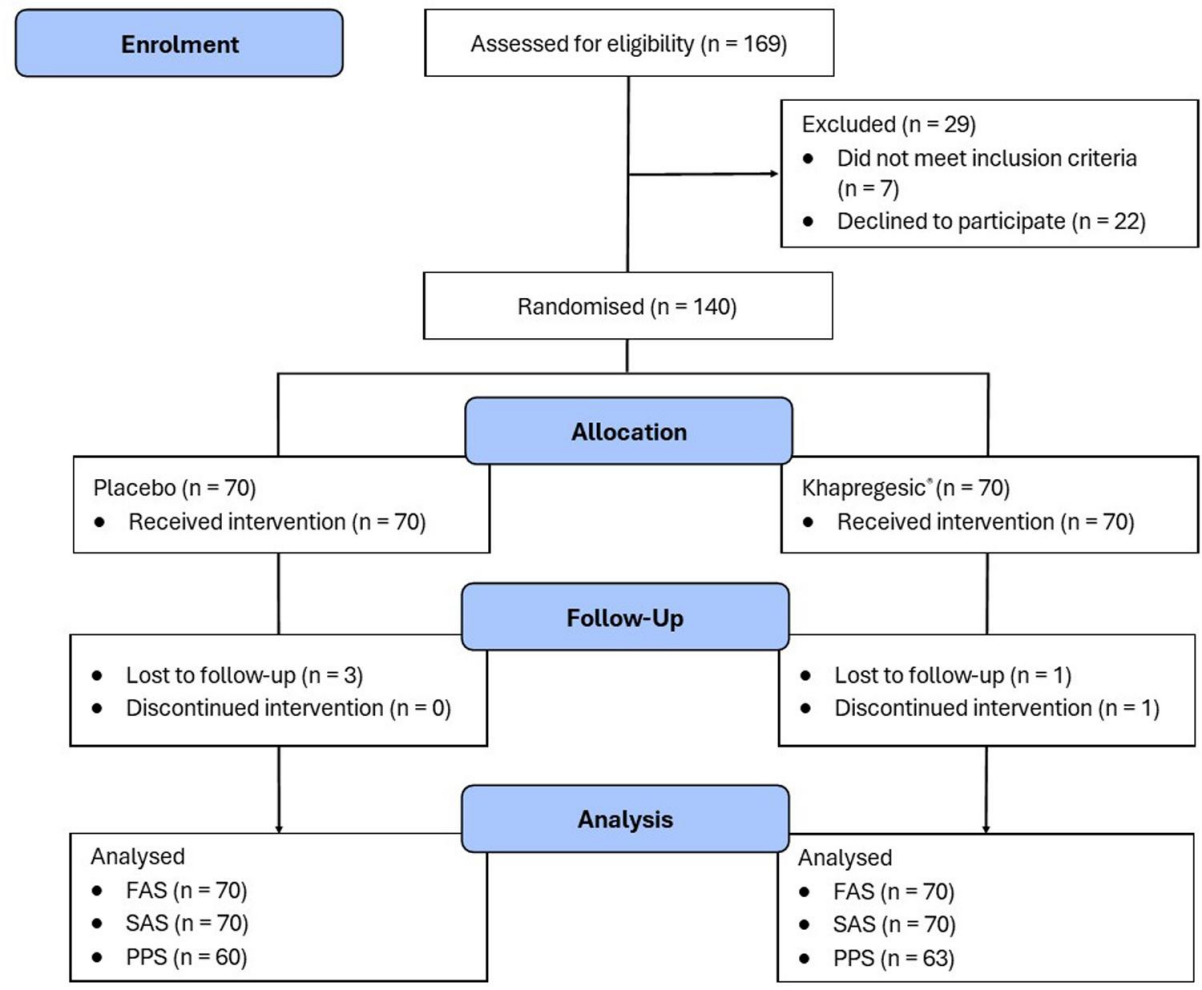
Systematic illustration of study design.

This was a two-arm, parallel-group, randomised, double-blind, placebo-controlled trial (Figure 1). Interested volunteers completed a screening survey, providing background information and details of menopausal-related symptoms. If deemed potentially eligible, they were contacted by a researcher and underwent a more comprehensive telephone interview. Further information was obtained about their menopausal symptoms and menstrual status. Moreover, other details were obtained regarding the eligibility criteria, including current treatments, medications, and physical and mental health history. A full explanation of the study was provided to participants, and, if eligible and willing to participate, they were required to sign an electronic version of the informed consent form. After completing the consent form, participants received their study tablets by express post. The day before starting the tablets, participants had a blood sample collected at a commercial blood collection centre, where safety assessments of liver and renal function and a full blood examination were undertaken. Participants took their study tablets (2 tablets twice daily) for 28 days, and a final blood collection was performed on day 28. Participants also completed self-report questions online at baseline (the day before starting the study tablets), day 14, and day 28.

### 2.2 Recruitment and randomisation

Recruitment for this study occurred from June 2025 to October 2025, using social media advertisements and promotion to a database of interested volunteers. Using a randomisation structure comprising 14 permuted blocks, with 10 participants per block, eligible participants were randomly assigned to one of two conditions (KS or placebo) in equal proportions using a randomisation calculator. The randomisation structure was undertaken by a study sponsor who was not involved in study recruitment, data collection, or data analysis. A participant number was designated based on the order of participant enrolment. All tablets were packed in identical containers, and the study sponsor held bottle codes. Researchers and the statistician were blind to the group allocation until all outcomes were collected, a blind review was completed, and the database was locked.

### 2.3 Participants

#### 2.3.1 Inclusion criteria

Inclusion criteria for the trial comprised the following: women aged 42–62 years; had an intact uterus and ovaries; reported the presence of menopausal/ climactic symptoms for at least 3 months; had a score greater than or equal to 12 on the Greene Climacteric Scale (GCS); had not experienced menses in the last 12 months, or for at least 3 months had experienced changes in the menstrual cycle (e.g., bleeding days, heaviness of bleeding, or days between menses); non-smoker; BMI between 18 and 30 kg/m^2^; had no plan to commence new interventions over the study period; and understood and was willing and able to comply with all study procedures.

#### 2.3.2 Exclusion criteria

The exclusion criteria comprised the following: had a recently diagnosed or unmanaged medical condition, including but not limited to diabetes, hypertension, cardiovascular disease, gastrointestinal disease, autoimmune disease, cancer, endocrine disease, or chronic/acute pain condition; had a diagnosis of a neurological or psychiatric condition, including but not limited to a psychiatric disorder (other than mild-to-moderate depression or anxiety), Parkinson's disease, or Alzheimer's disease; in the last 3 months, commenced, or changed pharmaceutical medications, nutritional, or herbal supplements likely to affect treatment outcomes or had an expectation to change during the study duration; planned to make major lifestyle changes in the next 2 months; had an alcohol intake greater than 14 standard drinks per week; had a current or 12-month history of regular illicit drug use; pregnant women, women who are breastfeeding, or women who planned to become pregnant during the study period; in the last year, had a significant surgery that affected their general health or daily function, or had a planned surgery in the next 2 months; or participated in any other clinical trial in the last month.

### 2.4 Interventions

The intervention comprised either Khapregesic® or a placebo. Khapregesic® is a Khaya senegalensis preparation derived from the dry stem bark. Participants were instructed to take 2 tablets twice daily (morning and evening), with or without food. Each Khapregesic® tablet contained 500 mg of Khapregesic®, delivering a total daily dose of 2 g. The active and placebo tablets were identical in appearance, matched in shape, colour, and size, and both contained similar excipients (calcium hydrogen phosphate dihydrate, colloidal anhydrous silica, croscarmellose sodium, crospovidone, glyceryl monostearate, hypromellose, macrogol, magnesium stearate, microcrystalline cellulose, and povidone). Adherence to tablet intake was assessed by asking participants to count the remaining tablets at the end of the study. Treatment blinding was evaluated by asking participants to predict their condition allocation (placebo, KS, or unsure) at the end of the study, along with a reason for their prediction.

### 2.5 Outcome measures

**Greene Climacteric Scale (GCS):** The GCS is a validated 21-item measure of menopause symptoms rated on a 4-point scale (not at all to extremely) experienced over the last month (20). It can be used to assess changes in different symptoms, before and after menopause treatment. Psychological, physical, and vasomotor subscores can be calculated, although the total score was established as the primary outcome measure.

**Hot Flush Rating Scale (HFRS):** The HFRS is a 5-item questionnaire assessing the frequency and problems associated with hot flushes over the past week (22).

**PROMIS Sleep Disturbance and Sleep-Related Impairment Scale (PROMIS Sleep):** The PROMIS Sleep is a validated self-report questionnaire that assesses sleep quality and sleep-related impairment over the last 7 days (23).

**Functional Assessment of Chronic Illness Therapy—Fatigue Scale (FACIT-Fatigue):** The FACIT-Fatigue is a validated 13-item measure that assesses self-reported fatigue and its impact on daily activities and function (24). Symptoms experienced over the last week are rated on a 5-point scale, ranging from “not at all” to “very much”.

**Patient Global Impression of Change (PGIC):** The PGIC reflects a person's belief about the efficacy of treatment and is widely used in clinical trials (25). Respondents are asked to estimate the difference between their current and previous health state based on a 7-point Likert scale ranging from “very much improved” to “very much worse.”

**Birmingham IBS Symptom Questionnaire (BISQ):** The BISQ is a validated, 11-item self-report questionnaire that assesses the frequency of a range of abdominal and bowel symptoms on a 6-point scale ranging from “All of the time” to “None of the time” range of digestive symptoms (26).

**Bristol Stool Chart (BSC):** The BSC is a diagnostic tool used to classify human faeces into 7 categories (27). It was administered fortnightly to examine changes in stool form over time. Participants were required to choose the overall stool type that they experienced over the past week.

#### 2.5.1 Safety measures

The tolerability of tablet intake was assessed using an online questionnaire enquiring about the experience of any adverse events. Moreover, at the end of the study, participants completed the Patient Global Assessment of Tolerability to Therapy (PGATT), in which they rated their tolerability to tablet intake on a scale from poor to excellent.

### 2.6 Sample size calculations

In a recently completed randomised, double-blind, placebo-controlled trial on Khapregesic® in women aged 18–50 years experiencing menstrual distress, effect sizes for the alleviation of symptoms such as pain, irritability, anxiety, and sleep disturbances ranged from approximately 0.35–0.5 (17). Therefore, an effect size of 0.45 was predicted. Assuming a power of 80% and a type one error rate (alpha) of 5%, the number of total participants required to find an effect is 124. Assuming a 10% dropout rate, it is planned to recruit 140 participants in total, which is hypothesised to provide sufficient power to detect an effect compared to placebo, even after dropouts.

### 2.7 Statistical analysis

Outcome analyses were conducted on the full analysis set (FAS), per-protocol set (PPS), and safety analysis set (SAS). In the FAS, an intention-to-treat analysis was undertaken, with all participants analysed in their originally assigned group, and for missing values, multiple imputation using the expectation-maximisation method was applied. Analyses were conducted on all participants, followed by separate analyses of perimenopausal and postmenopausal women. This subgroup analysis was prespecified, with an equal distribution of peri- and post-menopausal women recruited. Perimenopausal women were defined as women who have experienced changes in their menstrual cycle for at least 3 months, characterised by alterations in the days between cycles or changes in menstrual flow. Post-menopausal women were defined as women who have not had a period for 12 months or more. A univariate ANOVA assessed differences between intervention groups (placebo and KS) for treatment outcomes comprising the GCS, FACIT-Fatigue, PROMIS Sleep, BISQ, and HFRS scores. Differences in the change in scores from day 0 to day 28 were examined between the two intervention groups, with covariates including age, BMI, menopausal status, CTTES scores, and corresponding baseline values. Estimated means and standard errors are included in the relevant tables. Cohen's D effect sizes were calculated for each outcome measure. Analyses of HFRS were conducted only among participants who reported experiencing at least 1 hot flush or night sweat per week. To examine within-group changes over time, a repeated-measures ANOVA was used with the time points days 0, 14, and 28 included. The covariates age, BMI, menopausal status, and CTTES scores were included. Estimated means and standard errors are included in the relevant tables. Bristol stool types were categorised into ideal (types 3 and 4), constipation-like (types 1 and 2), and diarrhoea-like (types 5, 6, and 7). A Wilcoxon Signed Ranks Test was used to examine within-group changes in stool type at days 0 and 28. Group differences in PGIC and PGATT ratings were analysed using a Chi-square test. Changes in safety blood markers (complete blood count, liver function, and renal function) were conducted on the PPS. Between-group analyses were conducted using an independent-samples *t*-test to compare changes from day 0 to day 28. Within-group changes in blood markers were examined using a paired-samples *T*-test. All data were analysed using SPSS (version 31; IBM, Armonk, NY) and the critical *p*-value was set at *p* ≤ 0.05 (two-tailed) for all analyses. As there was only 1 *a priori* primary outcome measure, no adjustment to the *p*-value for multiple testing was undertaken. Moreover, no *p*-value adjustments were undertaken for the secondary and subgroup outcome measures. These secondary findings are intended to help guide planning for future trials, but given the increased risk of type 1 error, statistically significant secondary findings should be considered exploratory and require validation in future studies.

## 3 Results

### 3.1 Study population

A total of 374 people completed the online screening questionnaire, 169 underwent telephone screening, and 140 were randomised. Of the 169 participants in the telephone screening, the most common reason for exclusion was withdrawal of consent after the telephone interview (*n* = 22). A systematic illustration of the study design and study recruitment is included in Figure 1.

Baseline demographic and clinical characteristics are detailed in Table 1. The two groups were similarly matched in age, BMI, marital status, and educational levels. Baseline scores on outcome measures were also similar between the two groups.

**Table 1:** Baseline sociodemographic and clinical characteristics.

Sociodemographic and clinical characteristics		Placebo (*n* = 70)	KS (*n* = 70)
Age (years)	Mean	51.83	52.39
	SE	4.99	5.32
Number of children	N	2.31	1.93
	Mean	0.13	0.14
Height (m)	Mean	1.65	1.63
	SE	0.01	0.01
Weight (kg)	Mean	68.16	67.99
	SE	1.14	1.10
BMI (kg/m^2^)	Mean	24.99	25.44
	SE	0.37	0.36
Marital Status (n)	Single	15	27
	Married/ defacto	55	43
Menopausal status	Perimenopause	40	31
	Post-menopause	30	39
Education (n)	Secondary	30	30
	Tertiary	26	28
	Post-graduate	14	12
Exercise days per week	Mean	1.79	1.81
	SE	0.25	0.26
Occupation (n)	Professional	21	20
	Clerical support worker	12	10
	Elementary occupation	7	7
	Services and sales worker	4	7
	Manager	4	6
	Unemployed	3	1
	Craft & related trades worker	1	3
	Student	2	0
	Skilled agricultural, forestry and fishery worker	1	0
	Retired	0	1
	Armed forces occupations	0	1
	Technicians and associated trades	15	14
GCS Total Score—Screening	Mean	24.04	25.09
	SE	1.00	1.16
GCS Total Score—Day 0	Mean	20.53	22.06
	SE	0.91	0.91
BISQ score	Mean	8.49	9.47
	SE	0.74	0.79
FACIT Fatigue Score	Mean	19.39	18.60
	SE	1.21	1.17
HRFS: weekly hot flushes (n)	Mean	19.66	13.51
	SE	5.11	3.40
HRFS: weekly night sweats (n)	Mean	8.33	6.20
	SE	2.46	1.33
HFRS: problem rating	Mean	4.19	4.64
	SE	0.41	0.41
HFRS: distress rating	Mean	3.50	3.89
	SE	0.38	0.39
HFRS: interference rating	Mean	2.71	2.96
	SE	0.32	0.34
PROMIS Sleep: Sleep disturbance (T-score)	Mean	55.44	56.59
	SE	1.06	0.91
PROMIS Sleep: Sleep-related impairment (T-score)	Mean	58.93	58.80
	SE	0.92	0.84

### 3.2 Outcome measures

**Figure 2:**
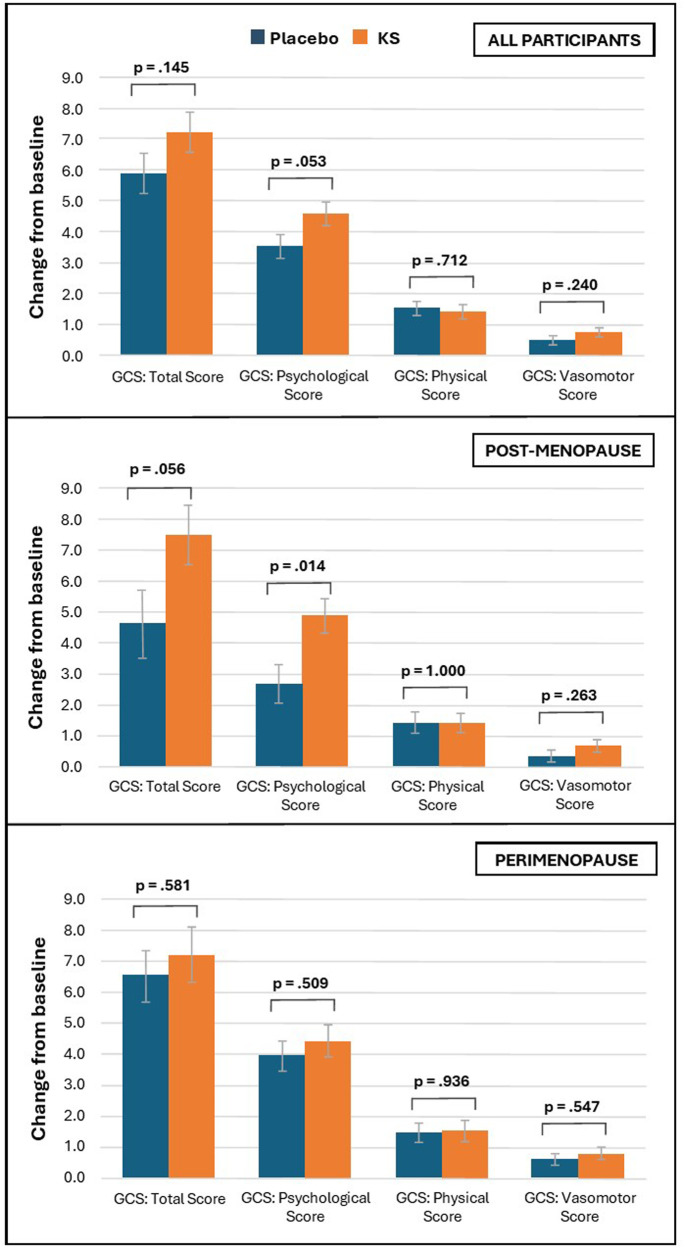
Change in GCS scores from day 0 to day 28 (vertical bars represent standard error) (full analysis set).

**GCS total and subscale scores:** As demonstrated in Table 2 and Figure 2, there was no statistically significant group difference in the change in the GCS total score (primary outcome measure) from day 0 to day 28 (*β*: 1.34; 95% CI: −0.47, 3.15; d = 0.25, *p* = 0.145). In the KS group, the mean total score decreased by 7.23 points (95% CI: 5.96, 8.50; *p* < 0.001), and in the placebo group, it decreased by 5.89 points (95% CI: 4.62, 7.16; *p* < 0.001). An analysis of the PPS revealed similar findings.

**Table 2:** Change in self-report measures from day 0 to day 28 (estimated marginal means) (FAS).

Outcome measures		Placebo (*n* = 70)					KS (*n* = 70)					*p*-value^b^	Cohen's D
		Day 0^a^	Day 14^a^	Day 28^a^	Change^b^	*P*-value^a^	Day 0^a^	Day 14^a^	Day 28^a^	Change^b^	*P*-value^a^		
GCS—Total Score	Mean	20.65	15.28	15.01	5.89	<.001	21.94	16.48	14.45	7.23	<.001	0.145	0.25
	SE	0.92	0.84	0.85	0.64		0.92	0.84	0.85	0.64			
GCS—Psychological Score	Mean	11.99	8.59	8.62	3.54	<.001	12.73	9.38	7.97	4.58	<.001	0.053	0.33
	SE	0.59	0.54	0.52	0.38		0.59	0.54	0.52	0.38			
GCS—Physical score	Mean	4.68	3.53	3.20	1.54	0.001	4.89	3.91	3.42	1.42	<.001	0.712	0.06
	SE	0.33	0.30	0.27	0.22		0.33	0.30	0.27	0.22			
GCS—Vasomotor score	Mean	2.37	1.77	1.88	0.51	0.005	2.48	1.67	1.71	0.75	<.001	0.240	0.21
	SE	0.19	0.17	0.18	0.14		0.19	0.17	0.18	0.14			
FACIT- Fatigue Score	Mean	19.46	12.85	12.33	6.90	<.001	18.52	13.41	11.17	7.59	<.001	0.512	0.11
	SE	1.18	1.08	0.94	0.74		1.18	1.08	0.94	0.74			
PROMIS Sleep Disturbance T-score	Mean	55.48	50.65	50.41	5.21	<.001	56.56	52.14	49.15	7.28	<.001	0.114	0.27
	SE	0.99	1.05	1.17	0.91		0.99	1.05	1.17	0.91			
PROMIS Sleep-Related Impairment T-score	Mean	59.12	55.66	54.46	4.60	<.001	58.61	54.56	53.38	5.29	<.001	0.566	0.10
	SE	0.88	1.00	1.07	0.84		0.88	1.00	1.07	0.84			
BISQ Score	Mean	8.51	6.54	5.99	2.76	<.001	9.45	7.36	6.64	2.57	0.003	0.809	0.04
	SE	0.76	0.68	0.66	0.54		0.76	0.68	0.66	0.54			

a *P*-values (within group) and estimated means are generated from repeated-measures ANOVAs adjusted for age, BMI, Menopausal status, and CTTES score.

b *P*-values and estimated means (change from D0 to D28) generated from univariate ANOVAs adjusted for age, BMI, menopausal status, CTTES score, and corresponding baseline values.

There was a near statistically significant group difference in the change in the GCS psychological score from day 0 to day 28 (*β*: 1.04; 95% CI: −0.01, 2.10; d = 0.33, *p* = 0.053). In the KS group, the mean psychological score decreased by 4.58 points (95% CI: 3.84, 5.33, *p* < 0.001) and in the placebo group, it decreased by 3.54 points (95% CI: 2.79, 4.28, *p* < 0.001). There were no statistically significant group differences in the change in GCS physical (*p* = 0.712) and vasomotor scores (*p* = 0.240) from day 0 to day 28. An analysis of the PPS revealed similar findings.

An analysis of the change in the GCS scores by menopausal status revealed a near-significant group difference in post-menopausal women on the total score (*β*: 2.88; 95% CI: −0.07, 5.83; d = 0.48, *p* = 0.056), a statistically significant group difference on the psychological score (*β*: 2.18; 95% CI: 0.47, 3.90; d = 0.63, *p* = 0.014), but no statistically significant group difference on physical (*p* = 1.000) or vasomotor (*p* = 0.263) scores (Figure 2 and Supplementary Table S1). Postmenopausal women in the KS group experienced a mean reduction of 4.89 points in the GCS psychological score (95% CI: 3.78, 6.00; *p* < 0.001), whereas scores in the placebo group decreased by 2.71 points (95% CI: 1.43, 3.98; *p* = 0.002). In peri-menopausal women, there were no group differences in changes in the GCS total (*p* = 0.581), psychological (*p* = 0.509), physical (*p* = 0.936), or vasomotor (*p* = 0.547) scores. An analysis of the PPS revealed similar findings.

**Figure 3:**
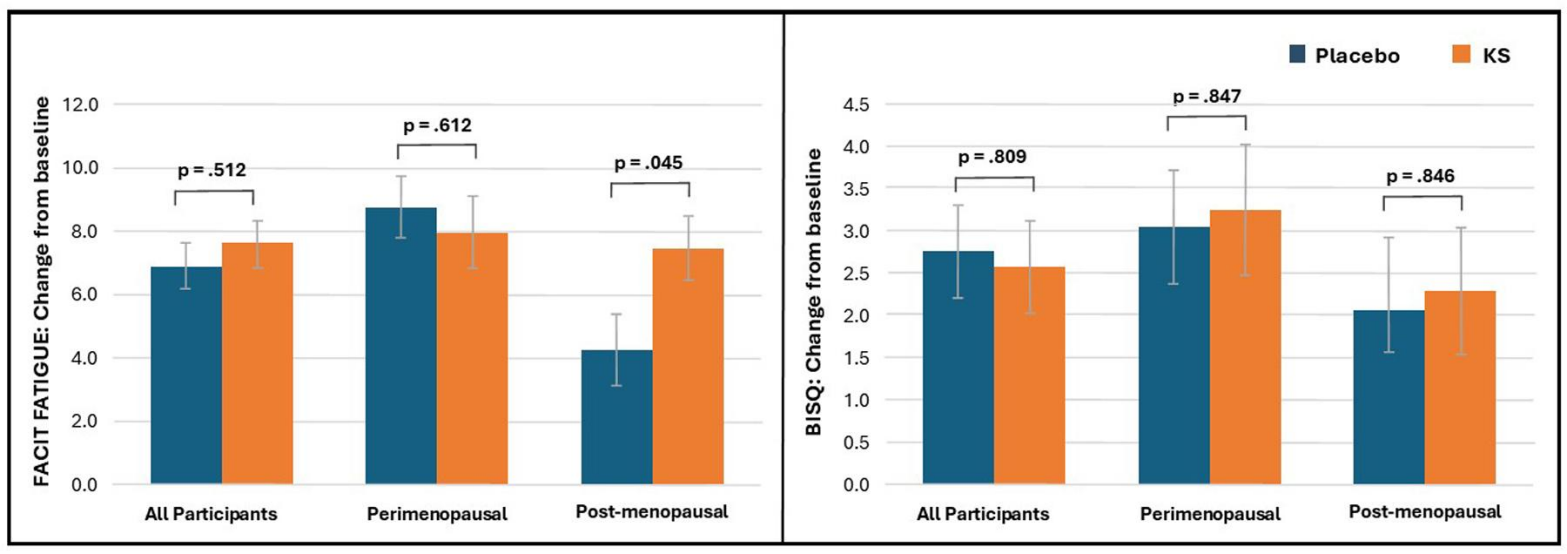
Change in FACIT Fatigue and BISQ scores from day 0 to day 28 (vertical bars represent standard error) (Full analysis set).

**FACIT Fatigue Score:** As demonstrated in Table 2 and Figure 3, there was no statistically significant group difference in the change in the FACIT fatigue score from day 0 to day 28 (*β*: 0.69; 95% CI: −1.38, 2.76; d = 0.11, *p* = 0.512). However, an analysis of the change in the FACIT fatigue score by menopausal status revealed a statistically significant group difference in post-menopausal women (*β*: 3.20; 95% CI: 0.07, 6.33; d = 0.50, *p* = 0.045), but no statistically significant group difference in perimenopausal women (*β*: 0.79; 95% CI: −3.88, 2.30; d = 0.12, *p* = 0.612) (Figure 3 and Supplementary Tables S1, 2). Postmenopausal women in the KS group experienced a mean reduction of 7.45 points (95% CI: 5.45, 9.45, *p* < 0.001), while scores in the placebo group decreased by 4.25 points (95% CI: 1.95, 6.55, *p* = 0.014). An analysis of the PPS revealed similar findings, although statistically significant group differences in the post-menopausal group no longer reached significance (*p* = 0.122).

**HFRS Scores:** As demonstrated in Supplementary Table S3, there were no group differences in changes in the number of hot flushes (*p* = 0.711), night sweats (*p* = 0.290), problem ratings (*p* = 0.366), distress ratings (*p* = 0.281), or interference ratings (*p* = 0.374). Moreover, analyses by menopausal status revealed no statistically significant group differences in changes in HFRS scores over time in either perimenopausal or postmenopausal women (Supplementary Tables S4, S5).

**Figure 4:**
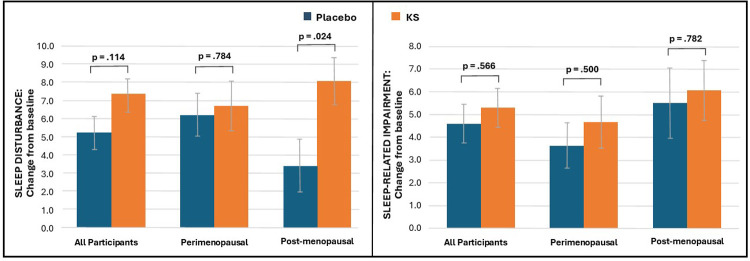
Change in PROMIS Sleep scores from day 0 to day 28 (vertical bars represent standard error) (Full analysis set).

**PROMIS Sleep Disturbance Score:** As demonstrated in Table 2 and Figure 4, there was no statistically significant group difference in the change in the PROMIS Sleep Disturbance T-score from day 0 to day 28 (*β*: 2.07; 95% CI: −0.50, 4.64; d = 0.27, *p* = 0.114). However, an analysis of the change in PROMIS Sleep Disturbance T-score by menopausal status revealed a statistically significant group difference in post-menopausal women (*β*: 4.66; 95% CI: 0.64, 8.68; d = 0.57, *p* = 0.024) (Figure 4 and Supplementary Table S1) but no statistically significant group difference in perimenopausal women (*β*: 0.51; 95% CI: −3.17, 4.19; d = 0.07, *p* = 0.784) (Figure 4 and Supplementary Table S2). Postmenopausal women in the KS group experienced a mean reduction of 8.07 points (95% CI: 5.53, 10.62, *p* < 0.001), while scores in the placebo group decreased by 3.42 points (95% CI: 0.48, 6.35, *p* = 0.033). An analysis of the PPS revealed similar findings, although statistically significant group differences in the post-menopausal group no longer reached significance (*p* = 0.063).

**PROMIS Sleep-Related Impairment Score:** As demonstrated in Table 2 and Figure 4, there were no statistically significant differences between groups in changes in the PROMIS sleep-related impairment score from day 0 to day 28 (*p* = 0.566). Moreover, there were no group differences when results were analysed in perimenopausal (*p* = 0.500) and post-menopausal (*p* = 0.782) women (Figure 4 and Supplementary Tables S1, S2).

**BISQ Score:** As demonstrated in Table 2 and Figure 3, there were no statistically significant differences between groups in changes in the BISQ score (*p* = 0.809). Moreover, there were no group differences when results were analysed in perimenopausal (*p* = 0.847) and post-menopausal (*p* = 0.846) women (Figure 3 and Supplementary Tables S1, S2).

**Bristol Stool Types:** As detailed in Supplementary Table S6, there were no significant changes in Bristol Stool type on day 0 or day 28 in the KS group (*p* = 0.724). On day 0, 64% of participants reported an ideal stool type (types 3 or 4), and on day 28, 61% did so. However, in the placebo group, there was a statistically significant change in reported stool type: 44% of participants had an ideal stool type at day 0, increasing to 61% at day 28 (*p* = 0.044).

**PGIC ratings:** There were no group differences at any time point for all participants, nor were there differences when analyses were stratified by menopausal status (Supplementary Table S7).

### 3.3 Treatment compliance and efficacy of participant blinding

Participants counted the remaining tablets in tablet bottles at the end of the study. Based on these details, 95% (*n* = 127) of participants who completed the study took over 80% of their tablets.

To assess the effectiveness of condition concealment during the trial, participants predicted their group allocation (i.e., placebo, KS, or unsure) at the end of the study. Group concealment was maintained, as 55% of participants in the placebo group and 72% in the KS group were unsure or incorrectly guessed their group allocation. Moreover, there was no difference in treatment prediction accuracy between the two groups (*p* = 0.093).

### 3.4 Adverse reactions and treatment discontinuation

Participants reported no serious adverse events, and the frequency of adverse events classified as possibly or probably related to tablet intake was similar (Table 3). In the KS group, 57 (81.4%) participants did not report an adverse reaction compared to 63 (90.0%) in the placebo group. There was a tendency for a greater number of gastrointestinal adverse reactions in the KS group compared to the placebo group (15.7% vs 7.1%). This comprised increased reports of loose stools, constipation, abdominal pain, and stomach bloating. Three participants (4.3%) in the KS group also reported experiencing headaches, compared to one participant (1.4%) in the placebo group. The PGATT ratings completed on day 28 showed that 92.7% of participants in the KS group reported good or excellent tolerability to the tablets, compared with 91.0% in the placebo group (Supplementary Table S8). Moderate tolerability to the IP was reported by 5 participants (7.4%) in the KS group compared to 6 participants (9.0%) in the placebo group. An analysis of changes in liver and renal function and full blood examinations revealed no significant changes over time (Supplementary Table S9).

**Table 3:** Possibly or probably related AEs by class and term.

AE Class	Diagnosis or symptom	Placebo (*n* = 70)	KS (*n* = 70)
Endocrine	**Number of participants**	**1** (**1.4%)**	**1** (**1.4%)**
	xerostomia (increased thirst)	1 (1.4%)	1 (1.4%)
Gastrointestinal	**Number of participants**	**5** (**7.1%)**	**11** (**15.7%)**
	Increased bowel movements/ loose stools	0 (0.0%)	3 (4.3%)
	Constipation	0 (0.0%)	2 (2.9%)
	Abdominal pain	2 (2.9%)	4 (5.7%)
	Stomach bloating	1 (1.4%)	4 (5.7%)
	Increased appetite	0 (0.0%)	0 (0.0%)
	Nausea	2 (2.9%)	1 (1.4%)
Neurological	**Number of participants**	**2** (**2.9%)**	**4** (**5.7%)**
	Headaches	1 (1.4%)	3 (4.3%)
	Worsened sleep	0 (0.0%)	1 (1.4%)
	Feeling hot	0 (0.0%)	1 (1.4%)
	Tiredness	1 (1.4%)	0 (0.0%)
Musculoskeletal	**Number of participants**	**0** (**0.0%)**	**1** (**1.4%)**
	Leg pain	0 (0.0%)	1 (1.4%)
Urological	**Number of participants**	**1** (**1.4%)**	**0** (**0.0%)**
	Increased urination	1 (1.4%)	0 (0.0%)
**Number of participants experiencing no treatment-related AEs**		**63 (90.0%)**	**57** (**81.4%)**

Note: Some participants experienced more than one treatment-related AE.

Bold text and numbers refer to the number of participants experiencing AEs, not the number of reported incidences. Some participants experienced more than one treatment-related AE.

## 4 Discussion

It is estimated that during the menopausal transition, up to 85% of women experience menopausal symptoms. While hormonal therapy is recommended for the treatment of menopausal symptoms, particularly vasomotor symptoms, holistic approaches are also encouraged. In this randomised, double-blind, placebo-controlled study, KS supplementation for 28 days demonstrated no group differences in any outcome measure comprising the GCS total score (primary outcome measure), GCS subscale scores, and other self-report measures of sleep, fatigue, hot flushes, and digestive health. This demonstrates that when data from the full cohort (peri- and postmenopausal women) are evaluated, KS supplementation had no beneficial effect compared to placebo. However, *a priori* subgroup analyses revealed KS had positive effects on psychological symptoms, fatigue, and sleep disturbances in post-menopausal women. These secondary subgroup findings in post-menopausal women should be interpreted cautiously due to an increased risk of type 1 error and require further investigation to validate. However, positive self-reported improvements across 3 separate outcome measures increase the robustness of the findings. Moreover, in post-menopausal women, KS supplementation resulted in a moderate-sized effect on psychological symptoms, fatigue, and sleep, as evidenced by moderate effect sizes of 0.63, 0.50, and 0.57, respectively.

KS supplementation for 28 days was generally well tolerated. There were no serious adverse reactions and no significant changes in liver or renal function or in full blood count. Moreover, 92.7% of participants in the KS group reported good or excellent tolerance to the tablets (compared with 91.0% in the placebo group). However, there was a tendency for a greater number of gastrointestinal adverse reactions in the KS group compared to the placebo group (15.7% vs. 7.1%). This comprised increased reports of loose stools, constipation, abdominal pain, and stomach bloating, which resolved over time. This is consistent with observational data where some women have reported mild, transient gastrointestinal symptoms early in treatment, which naturally resolve over time. An examination of the effects of KS on gastrointestinal symptoms is important, as KS has been shown to inhibit cyclooxygenase (COX) type 2 enzymes (18, 19, 28). COX inhibitors, such as non-steroidal anti-inflammatory drugs, are often associated with gastrointestinal symptoms (29), and the mild, transient effects of KS on gastrointestinal symptoms are encouraging. However, given the short duration of the study, longer trials are required to validate the safety and tolerability of KS supplementation beyond 28 days.

The inconsistent findings between post-menopausal and perimenopausal women require further investigation. Preclinical studies suggest KS has anti-inflammatory (18, 19, 28) and antioxidant (19, 20) effects. In an animal trial, KS also influenced gamma-aminobutyric acid neurotransmission (13). As mood disturbances, poor sleep, and fatigue are associated with disturbances in these physiological mechanisms, they are possible mechanisms of action of KS on these health-related outcomes. However, as perimenopause is associated with significant fluctuations in sex hormones, compared to more stable, albeit lower concentrations of sex hormones in post-menopausal women, different treatment regimens may be required. This may include higher doses or a longer treatment duration in perimenopausal women. The administration of KS in conjunction with pharmacological and nonpharmacological interventions may also be associated with greater treatment efficacy.

### 4.1 Strengths, limitations and directions for future research

The positive effects of KS supplementation on psychological symptoms in post-menopausal women are encouraging but should be considered exploratory. In a previous study of women experiencing menstrual distress, improvement in emotional symptoms was identified (17). Given these findings, further investigation into its mood-enhancing effects is recommended. Epidemiological data consistently show that women are prescribed antidepressants and anxiolytics approximately twice as often as men, often for distress, fatigue, and sleep disturbance rather than for clearly defined major depressive or anxiety disorders (30, 31). However, psychotropic use can be associated with adverse reactions such as insomnia, somnolence, fatigue, weight gain, and cognitive deficits (32–34). These are symptoms that typically worsen and are associated with significant distress during the menopausal transition.

The positive effects of KS on fatigue and sleep disturbances in post-menopausal women make it a promising option for the alleviation of these troublesome symptoms, with an encouraging safety profile. Although not investigated in this study, the pain-relieving effects of KS in post-menopausal women are also worthy of investigation, particularly as musculoskeletal pain is reported by more than 70% of women during the menopausal transition (35, 36). Positive pain-relieving effects were previously identified in women experiencing menstrual distress (17).

Longer studies are necessary to determine the long-term safety and efficacy profile of KS, as the clinical trials conducted with Khapregesic® have been 1 month in duration. Moreover, longer trials will also be important for determining whether KS can affect vasomotor symptoms. This is particularly relevant, as hormonal therapies are administered for longer periods, with optimal symptom alleviation often occurring after 3 months (37, 38). The mechanisms of action of KS supplementation were not investigated in this study. Therefore, further research is required to help understand its biological actions. This includes an investigation into its effects on sex hormones, markers of inflammation and oxidative stress, and its influence on neurotransmitter activity. Finally, as only self-report outcome measures were utilised in this study, which are subject to reporting bias, additional outcomes should be considered, including diaries, daily symptom monitoring, clinician-administered measures, and other objective measures.

## 5 Conclusions

In summary, in the full cohort of participants comprising both peri- and post-menopausal women, this study demonstrated KS had no beneficial effect on menopausal-related symptoms compared with the placebo. However, secondary analyses revealed that while 28-day supplementation with KS had no positive effect in peri-menopausal women, therapeutic effects were identified in post-menopausal women on menopausal-related psychological symptoms, fatigue, and sleep. Given the increased risk of type 1 error due to multiple analyses, these findings should be considered exploratory and require confirmation in future trials. Moreover, trials lasting longer than 28 days, using varying treatment regimens, and examining potential mechanistic actions of KS will be important.

## Data Availability

The raw data supporting the conclusions of this article will be made available by the authors, without undue reservation.
