# Supplementary material for: An examination into the safety and efficacy of Khapregesic®, a Khaya senegalensis preparation, on physical and psychological wellbeing in women experiencing menopausal symptoms: a randomised, double-blind, placebo-controlled trial
Source: Front Reprod Health. 2026 Jun 2;8:1824321. doi: 10.3389/frph.2026.1824321 (PMC13269224; doi:10.3389/frph.2026.1824321)
Supplement: Supplementary file 1 [file Table1.docx]

# **Supplementary File:**

## An examination into the safety and efficacy of Khapregesic®, a Khaya senegalensis preparation, on physical and psychological wellbeing in women experiencing menopausal symptoms: a randomised, double-blind, placebo-controlled trial

**Author names:**

^1,2^ Adrian L Lopresti - ORCID iD: 0000-0002-6409-7839

^1^ Stephen J Smith - ORCID iD: 0000-0002-3875-4815

^3^Frederick R Ferdinands – ORCID iD: 0009-0007-3912-8596

**Author affiliations:**

^1^Clinical Research Australia, Perth, Western Australia, 6023, Australia

^2^College of Science, Health, Engineering and Education, Murdoch University, Perth, Western Australia, 6150, Australia

^3^Bioactive Natural Health Pty Ltd, Perth, Western Australia, 6014, Australia

**Corresponding author:** Adrian Lopresti, Clinical Research Australia, 38 Arnisdale Road, Duncraig, Western Australia 6023, [adrian@clinicalresearch.com.au](mailto:adrian@clinicalresearch.com.au)

## Supplementary Table 1. Post-menopausal women: Change in self-report measures from day 0 to day 28 (estimated marginal means) (FAS)

|  | | Placebo (n = 30) | | | | | KS (n = 39) | | | | | p-value^b^ | Cohen's D |
| --- | --- | --- | --- | --- | --- | --- | --- | --- | --- | --- | --- | --- | --- |
|  |  | Day 0^a^ | Day 14^a^ | Day 28^a^ | Change^b^ | P-value^a^ | Day 0^a^ | Day 14^a^ | Day 28^a^ | Change^b^ | P-value^a^ |  |  |
| GCS - Total Score | Mean | 20.84 | 15.34 | 16.69 | 4.63 | 0.001 | 23.10 | 17.30 | 15.23 | 7.51 | < .001 | 0.056 | 0.48 |
|  | SE | 1.42 | 1.19 | 1.40 | 1.09 |  | 1.24 | 1.04 | 1.22 | 0.95 |  |  |  |
| GCS - Psychological Score | Mean | 11.85 | 8.41 | 9.37 | 2.71 | 0.002 | 12.81 | 9.33 | 7.74 | 4.89 | < .001 | 0.014 | 0.63 |
|  | SE | 0.99 | 0.85 | 0.85 | 0.64 |  | 0.86 | 0.74 | 0.74 | 0.56 |  |  |  |
| GCS - Physical score | Mean | 4.82 | 3.41 | 3.55 | 1.45 | 0.004 | 5.42 | 4.30 | 3.84 | 1.45 | < .001 | 1.000 | 0.00 |
|  | SE | 0.48 | 0.37 | 0.41 | 0.35 |  | 0.42 | 0.33 | 0.36 | 0.31 |  |  |  |
| GCS - Vasomotor score | Mean | 2.56 | 2.03 | 2.24 | 0.35 | 0.176 | 2.80 | 1.95 | 2.08 | 0.69 | 0.001 | 0.263 | 0.28 |
|  | SE | 0.29 | 0.28 | 0.31 | 0.22 |  | 0.26 | 0.25 | 0.27 | 0.19 |  |  |  |
| FACIT- Fatigue Score | Mean | 16.56 | 11.36 | 13.18 | 4.25 | 0.014 | 20.21 | 14.65 | 12.09 | 7.45 | < .001 | 0.045 | 0.50 |
|  | SE | 1.72 | 1.57 | 1.51 | 1.15 |  | 1.51 | 1.37 | 1.32 | 1.00 |  |  |  |
| BISQ Score | Mean | 8.27 | 6.71 | 6.53 | 2.06 | 0.102 | 9.31 | 7.23 | 6.77 | 2.29 | 0.007 | 0.846 | 0.05 |
|  | SE | 1.12 | 1.03 | 0.99 | 0.86 |  | 0.97 | 0.90 | 0.86 | 0.75 |  |  |  |
| PROMIS Sleep Disturbance T-score | Mean | 53.75 | 50.02 | 50.63 | 3.42 | 0.033 | 58.04 | 53.09 | 49.74 | 8.07 | < .001 | 0.024 | 0.57 |
|  | SE | 1.38 | 1.53 | 1.87 | 1.47 |  | 1.21 | 1.34 | 1.63 | 1.27 |  |  |  |
| PROMIS Sleep-Related Impairment T-score | Mean | 59.24 | 55.35 | 53.93 | 5.51 | 0.001 | 60.57 | 56.06 | 54.35 | 6.07 | < .001 | 0.782 | 0.07 |
|  | SE | 1.18 | 1.45 | 1.72 | 1.50 |  | 1.03 | 1.27 | 1.50 | 1.31 |  |  |  |

^a^ P-values (within group) and estimated means are generated from repeated-measures ANOVAs adjusted for age, BMI, Menopausal status, and CTTES score.

^b^ P-values and estimated means (change from D0 to D28) generated from univariate ANOVAs adjusted for age, BMI, menopausal status, CTTES score, and corresponding baseline value.

## Supplementary Table 2. Perimenopausal women: Change in self-report measures from day 0 to day 28 (estimated marginal means) (FAS)

|  | | Placebo (n = 40) | | | | | KS (n = 31) | | | | | p-value^b^ | Cohen's D |
| --- | --- | --- | --- | --- | --- | --- | --- | --- | --- | --- | --- | --- | --- |
|  |  | Day 0^a^ | Day 14^a^ | Day 28^a^ | Change^b^ | P-value^a^ | Day 0^a^ | Day 14^a^ | Day 28^a^ | Change^b^ | P-value^a^ |  |  |
| GCS - Total Score | Mean | 20.53 | 15.62 | 13.94 | 6.57 | < .001 | 20.45 | 14.96 | 13.24 | 7.23 | < .001 | 0.581 | 0.14 |
|  | SE | 1.22 | 1.16 | 1.04 | 0.77 |  | 1.39 | 1.32 | 1.19 | 0.88 |  |  |  |
| GCS - Psychological Score | Mean | 12.25 | 9.12 | 8.30 | 3.98 | < .001 | 12.42 | 8.95 | 7.94 | 4.44 | < .001 | 0.509 | 0.16 |
|  | SE | 0.73 | 0.67 | 0.63 | 0.45 |  | 0.83 | 0.76 | 0.72 | 0.52 |  |  |  |
| GCS - Physical score | Mean | 4.49 | 3.63 | 2.96 | 1.49 | < .001 | 4.33 | 3.40 | 2.85 | 1.53 | 0.001 | 0.936 | 0.02 |
|  | SE | 0.48 | 0.46 | 0.37 | 0.29 |  | 0.55 | 0.52 | 0.42 | 0.34 |  |  |  |
| GCS - Vasomotor score | Mean | 2.21 | 1.58 | 1.54 | 0.64 | 0.002 | 2.08 | 1.30 | 1.31 | 0.81 | 0.002 | 0.547 | 0.15 |
|  | SE | 0.26 | 0.19 | 0.22 | 0.18 |  | 0.29 | 0.22 | 0.25 | 0.20 |  |  |  |
| FACIT- Fatigue Score | Mean | 21.77 | 14.12 | 11.75 | 8.74 | < .001 | 16.23 | 11.65 | 9.93 | 7.95 | < .001 | 0.612 | 0.12 |
|  | SE | 1.62 | 1.52 | 1.23 | 0.99 |  | 1.84 | 1.73 | 1.40 | 1.13 |  |  |  |
| BISQ Score | Mean | 8.96 | 6.88 | 5.98 | 3.04 | 0.001 | 9.28 | 6.93 | 5.95 | 3.24 | 0.001 | 0.847 | 0.05 |
|  | SE | 1.08 | 0.90 | 0.86 | 0.67 |  | 1.23 | 1.03 | 0.98 | 0.77 |  |  |  |
| PROMIS Sleep Disturbance T-score | Mean | 56.69 | 51.02 | 50.22 | 6.21 | < .001 | 54.81 | 51.07 | 48.43 | 6.72 | < .001 | 0.784 | 0.07 |
|  | SE | 1.41 | 1.48 | 1.53 | 1.20 |  | 1.61 | 1.69 | 1.74 | 1.36 |  |  |  |
| PROMIS Sleep-Related Impairment T-score | Mean | 58.99 | 56.03 | 55.06 | 3.63 | < .001 | 56.19 | 52.50 | 51.89 | 4.68 | 0.001 | 0.500 | 0.16 |
|  | SE | 1.28 | 1.39 | 1.39 | 1.00 |  | 1.46 | 1.59 | 1.58 | 1.14 |  |  |  |

^a^ P-values (within group) and estimated means are generated from repeated-measures ANOVAs adjusted for age, BMI, Menopausal status, and CTTES score.

^b^ P-values and estimated means (change from D0 to D28) generated from univariate ANOVAs adjusted for age, BMI, menopausal status, CTTES score, and corresponding baseline value.

## Supplementary Table 3. Change in HFRS measures from day 0 to day 28 (estimated marginal means) (FAS)

|  | | Placebo (n = 50) | | | | | KS (n = 48) | | | | | p-value^b^ | Cohen's D |
| --- | --- | --- | --- | --- | --- | --- | --- | --- | --- | --- | --- | --- | --- |
|  |  | Day 0^a^ | Day 14^a^ | Day 28^a^ | Change^b^ | P-value^a^ | Day 0^a^ | Day 14^a^ | Day 28^a^ | Change^b^ | P-value^a^ |  |  |
| HFRS: hot flushes (n) | Mean | 21.27 | 12.94 | 10.84 | 11.47 | 0.029 | 24.84 | 12.85 | 10.63 | 13.14 | 0.004 | 0.711 | 0.08 |
|  | SE | 5.98 | 4.25 | 3.97 | 3.14 |  | 6.10 | 4.34 | 4.05 | 3.21 |  |  |  |
| HFRS: night sweats (n) | Mean | 12.04 | 11.24 | 7.64 | 3.24 | 0.016 | 7.38 | 4.58 | 3.55 | 5.05 | 0.039 | 0.290 | 0.22 |
|  | SE | 2.66 | 3.00 | 1.75 | 1.18 |  | 2.72 | 3.06 | 1.79 | 1.21 |  |  |  |
| HFRS: problem rating | Mean | 6.18 | 4.84 | 4.28 | 1.76 | < .001 | 5.44 | 4.04 | 3.36 | 2.23 | < .001 | 0.366 | 0.19 |
|  | SE | 0.38 | 0.41 | 0.42 | 0.36 |  | 0.39 | 0.42 | 0.43 | 0.37 |  |  |  |
| HFRS: distress rating | Mean | 5.08 | 3.76 | 3.40 | 1.59 | < .001 | 4.63 | 3.25 | 2.67 | 2.06 | < .001 | 0.281 | 0.22 |
|  | SE | 0.40 | 0.38 | 0.38 | 0.30 |  | 0.41 | 0.39 | 0.39 | 0.31 |  |  |  |
| HFRS: interference rating | Mean | 3.84 | 2.68 | 2.81 | 0.96 | 0.003 | 3.48 | 2.35 | 2.22 | 1.34 | < .001 | 0.374 | 0.18 |
|  | SE | 0.37 | 0.35 | 0.36 | 0.30 |  | 0.38 | 0.36 | 0.37 | 0.30 |  |  |  |

^a^ P-values (within group) and estimated means are generated from repeated-measures ANOVAs adjusted for age, BMI, Menopausal status, and CTTES score.

^b^ P-values and estimated means (change from D0 to D28) generated from univariate ANOVAs adjusted for age, BMI, menopausal status, CTTES score, and corresponding baseline value.

## Supplementary Table 4. Perimenopausal women: Change in HFRS measures from day 0 to day 28 (estimated marginal means) (FAS)

|  | | Placebo (n = 27) | | | | | KS (n = 22) | | | | | p-value^b^ | Cohen's D |
| --- | --- | --- | --- | --- | --- | --- | --- | --- | --- | --- | --- | --- | --- |
|  |  | Day 0^a^ | Day 14^a^ | Day 28^a^ | Change^b^ | P-value^a^ | Day 0^a^ | Day 14^a^ | Day 28^a^ | Change^b^ | P-value^a^ |  |  |
| HFRS: hot flushes (n) | Mean | 16.30 | 7.12 | 7.46 | 9.97 | 0.074 | 23.36 | 16.80 | 13.84 | 8.13 | 0.084 | 0.773 | 0.08 |
|  | SE | 7.32 | 5.42 | 6.23 | 4.13 |  | 8.14 | 6.03 | 6.93 | 4.59 |  |  |  |
| HFRS: night sweats (n) | Mean | 8.38 | 6.56 | 4.49 | 3.58 | 0.066 | 7.21 | 4.81 | 5.17 | 2.43 | 0.378 | 0.629 | 0.14 |
|  | SE | 2.32 | 1.94 | 1.78 | 1.54 |  | 2.58 | 2.16 | 1.98 | 1.71 |  |  |  |
| HFRS: problem rating | Mean | 6.23 | 5.46 | 4.30 | 1.71 | 0.001 | 4.81 | 4.11 | 3.59 | 1.50 | 0.043 | 0.794 | 0.08 |
|  | SE | 0.50 | 0.57 | 0.59 | 0.51 |  | 0.56 | 0.63 | 0.66 | 0.57 |  |  |  |
| HFRS: distress rating | Mean | 5.45 | 4.44 | 3.58 | 1.69 | < .001 | 4.31 | 3.41 | 2.83 | 1.70 | 0.006 | 0.998 | 0.00 |
|  | SE | 0.52 | 0.53 | 0.54 | 0.43 |  | 0.58 | 0.59 | 0.60 | 0.48 |  |  |  |
| HFRS: interference rating | Mean | 4.13 | 3.12 | 2.69 | 1.36 | 0.001 | 3.34 | 2.63 | 2.24 | 1.19 | 0.018 | 0.776 | 0.08 |
|  | SE | 0.47 | 0.55 | 0.53 | 0.39 |  | 0.52 | 0.62 | 0.59 | 0.44 |  |  |  |

^a^ P-values (within group) and estimated means are generated from repeated-measures ANOVAs adjusted for age, BMI, Menopausal status, and CTTES score.

^b^ P-values and estimated means (change from D0 to D28) generated from univariate ANOVAs adjusted for age, BMI, CTTES score, and corresponding baseline value.

## Supplementary Table 5. Post-menopausal women: Change in HFRS measures from day 0 to day 28 (estimated marginal means) (FAS)

|  | | Placebo (n = 23) | | | | | KS (n = 26) | | | | | p-value^b^ | Cohen's D |
| --- | --- | --- | --- | --- | --- | --- | --- | --- | --- | --- | --- | --- | --- |
|  |  | Day 0^a^ | Day 14^a^ | Day 28^a^ | Change^b^ | P-value^a^ | Day 0^a^ | Day 14^a^ | Day 28^a^ | Change^b^ | P-value^a^ |  |  |
| HFRS: hot flushes (n) | Mean | 25.90 | 17.38 | 13.96 | 12.44 | 0.184 | 27.16 | 11.63 | 8.66 | 18.07 | 0.031 | 0.366 | 0.27 |
|  | SE | 10.45 | 6.90 | 5.09 | 4.36 |  | 9.79 | 6.46 | 4.77 | 4.09 |  |  |  |
| HFRS: night sweats (n) | Mean | 15.40 | 15.91 | 10.62 | 2.90 | 0.140 | 8.34 | 5.12 | 2.80 | 7.20 | 0.070 | 0.105 | 0.48 |
|  | SE | 5.22 | 6.10 | 3.16 | 1.83 |  | 4.89 | 5.71 | 2.96 | 1.72 |  |  |  |
| HFRS: problem rating | Mean | 6.04 | 4.10 | 4.20 | 1.84 | 0.004 | 6.05 | 3.99 | 3.21 | 2.84 | < .001 | 0.208 | 0.37 |
|  | SE | 0.61 | 0.63 | 0.64 | 0.55 |  | 0.57 | 0.59 | 0.60 | 0.52 |  |  |  |
| HFRS: distress rating | Mean | 4.88 | 3.00 | 3.13 | 1.69 | 0.003 | 4.69 | 3.08 | 2.57 | 2.16 | < .001 | 0.479 | 0.21 |
|  | SE | 0.65 | 0.57 | 0.56 | 0.46 |  | 0.61 | 0.53 | 0.53 | 0.43 |  |  |  |
| HFRS: interference rating | Mean | 3.65 | 2.31 | 2.91 | 0.68 | 0.210 | 3.47 | 2.00 | 2.23 | 1.29 | 0.027 | 0.365 | 0.27 |
|  | SE | 0.60 | 0.47 | 0.53 | 0.47 |  | 0.56 | 0.44 | 0.50 | 0.44 |  |  |  |

^a^ P-values (within group) and estimated means are generated from repeated-measures ANOVAs adjusted for age, BMI, Menopausal status, and CTTES score.

^b^ P-values and estimated means (change from D0 to D28) generated from univariate ANOVAs adjusted for age, BMI, CTTES score, and corresponding baseline value.

##

## Supplementary Table 6. Frequency of Reported Bristol Stool Types

|  | Placebo | | | KS | | |
| --- | --- | --- | --- | --- | --- | --- |
|  | Day 0 | Day 28 | p-value | Day 0 | Day 28 | p-value |
| Ideal Stool type  (types 3 and 4) | 31 | 41 | 0.044 | 45 | 41 | 0.724 |
| Constipation-like  (types 1 and 2) | 21 | 13 |  | 12 | 16 |  |
| Diarrhoea-like  (types 5, 6, and 7) | 18 | 13 |  | 13 | 11 |  |

* Wilcoxon Signed Ranks Test

## Supplementary Table 7. Frequency of PGIC Responses at Days 14 and 28

| **All Participants** | | | | | | |
| --- | --- | --- | --- | --- | --- | --- |
| **Day 14** | | | | | | |
|  | Placebo | | KS | | p-value* | |
| Very Much Improved | 2 (2.9%) | | 1 (1.4%) | | 0.477 | |
| Much improved | 5 (7.2%) | | 7 (10.1%) | |  |  |
| Minimally improved | 21 (30.4%) | | 27 (39.1%) | |  |  |
| No change | 36 (52.2%) | | 27 (39.1%) | |  |  |
| Minimally worse | 4 (5.8%) | | 7 (10.1%) | |  |  |
| Much worse | 1 (1.4%) | | 0 (0.0%) | |  |  |
| **Day 28** | | | | | | |
|  | Placebo | | KS | | p-value* | |
| Very Much Improved | 2 (3.0%) | | 3 (4.4%) | | 0.610 | |
| Much improved | 10 (14.9%) | | 9 (13.2%) | |  |  |
| Minimally improved | 22 (32.8%) | | 30 (44.1%) | |  |  |
| No change | 28 (41.8%) | | 21 (30.9%) | |  |  |
| Minimally worse | 5 (7.5%) | | 4 (5.9%) | |  |  |
| Much worse | 0 (0.0%) | | 1 (1.5%) | |  |  |
| **Perimenopausal women** | | | | | | |
| **Day 14** | | | | | | |
|  | Placebo | | KS | | p-value* | |
| Very Much Improved | 2 (5.1%) | | 1 (3.3%) | | 0.695 | |
| Much improved | 3 (7.7%) | | 5 (16.7%) | |  |  |
| Minimally improved | 13 (33.3%) | | 12 (40.0%) | |  |  |
| No change | 18 (46.2%) | | 10 (33.3%) | |  |  |
| Minimally worse | 3 (7.7%) | | 2 (6.7%) | |  |  |
| Much worse | 0 (0.0%) | | 0 (0.0%) | |  |  |
| **Day 28** | | | | | | |
|  | Placebo | | KS | | p-value* | |
| Very Much Improved | 1 (2.7%) | | 3 (10.0%) | | 0.744 | |
| Much improved | 6 (16.2%) | | 6 (20.0%) | |  |  |
| Minimally improved | 14 (37.8%) | | 10 (33.3%) | |  |  |
| No change | 14 (37.8%) | | 10 (33.3%) | |  |  |
| Minimally worse | 2 (5.4%) | | 1 (3.3%) | |  |  |
| Much worse | 0 (0.0%) | | 0 (0.0%) | |  |  |
| **Postmenopausal women** | | | | | | |
| **Day 14** | | | | | | |
|  | | Placebo | | KS | | p-value* |
| Very Much Improved | | 0 (0.0%) | | 0 (0.0%) | | 0.316 |
| Much improved | | 2 (6.7%) | | 2 (5.1%) | |  |
| Minimally improved | | 8 (26.7%) | | 15 (38.5%) | |  |
| No change | | 18 (60.0%) | | 17 (43.6%) | |  |
| Minimally worse | | 1 (3.3%) | | 5 (12.8%) | |  |
| Much worse | | 1 (3.3%) | | 0 (0.0%) | |  |
| **Day 28** | | | | | | |
|  | | Placebo | | KS | | p-value* |
| Very Much Improved | | 1 (3.3%) | | 0 (0.0%) | | 0.236 |
| Much improved | | 4 (13.3%) | | 3 (7.9%) | |  |
| Minimally improved | | 8 (26.7%) | | 20 (52.6%) | |  |
| No change | | 14 (46.7%) | | 11 (28.9%) | |  |
| Minimally worse | | 3 (10.0%) | | 3 (7.9%) | |  |
| Much worse | | 0 (0.0%) | | 0 (0.0%) | |  |

* Chi-square test

## Supplementary Table 8. Frequency of PGATT Responses at Day 28

|  | **Placebo** | **KS** | **P-value*** |
| --- | --- | --- | --- |
| EXCELLENT. I experienced no discomfort or adverse effects | 49 (73.1%) | 45 (66.2%) | 0.483 |
| GOOD. I experienced minimal discomfort/ side effects, but it did not interfere with my normal activities | 12 (17.9%) | 18 (26.5%) |  |
| MODERATE. I experienced moderate discomfort/ side effects, and it had some effect on my normal activities | 6 (9.0%) | 5 (7.4%) |  |

* Chi-square test

## Supplementary Table 9. Change in blood concentrations over time (PPS)

|  | | **Placebo** | | | **KS** | | | **p-value^b^** | **Reference Range** |
| --- | --- | --- | --- | --- | --- | --- | --- | --- | --- |
|  |  | **Day 0** | **Day 28** | **p-value^a^** | **Day 0** | **Day 28** | **p-value^a^** |  |  |
| **Full blood count** | | | | | | | | | |
| Haemoglobin (g/L | N | 60 | 60 | 0.274 | 63 | 63 | 0.072 | 0.660 | 115 - 160 |
|  | Mean | 132.72 | 131.83 |  | 132.44 | 131.08 |  |  |  |
|  | SE | 1.06 | 1.09 |  | 0.95 | 0.96 |  |  |  |
| RCC (x10 12/L) | N | 60 | 60 | 0.331 | 63 | 63 | 0.430 | 0.303 | 3.80 - 5.80 |
|  | Mean | 4.40 | 4.99 |  | 4.39 | 4.38 |  |  |  |
|  | SE | 0.04 | 4.64 |  | 0.04 | 0.04 |  |  |  |
| PCV (x10 12/L) | N | 60 | 60 | 0.436 | 63 | 63 | 0.971 | 0.600 | 0.37 - 0.47 |
|  | Mean | 0.401 | 0.391 |  | 0.406 | 0.405 |  |  |  |
|  | SE | 0.012 | 0.060 |  | 0.009 | 0.009 |  |  |  |
| MCHC (g/L | N | 60 | 60 | 0.244 | 63 | 63 | 0.755 | 0.586 | 315 - 360 |
|  | Mean | 333.13 | 331.90 |  | 329.25 | 330.75 |  |  |  |
|  | SE | 1.08 | 1.06 |  | 4.93 | 0.86 |  |  |  |
| MCV (fL | N | 60 | 60 | 0.643 | 63 | 63 | 0.155 | 0.201 | 80 - 98 |
|  | Mean | 90.85 | 90.73 |  | 90.52 | 90.83 |  |  |  |
|  | SE | 0.44 | 0.41 |  | 0.51 | 0.54 |  |  |  |
| MCH (pg) | N | 60 | 60 | 0.192 | 63 | 63 | 0.005 | 0.353 | 27.0 - 34.0 |
|  | Mean | 30.22 | 29.57 |  | 30.21 | 30.01 |  |  |  |
|  | SE | 0.18 | 0.52 |  | 0.18 | 0.18 |  |  |  |
| RDW (%) | N | 60 | 60 | 0.250 | 63 | 63 | 0.714 | 0.640 | < 16 |
|  | Mean | 12.83 | 12.91 |  | 12.81 | 12.84 |  |  |  |
|  | SE | 0.10 | 0.12 |  | 0.11 | 0.09 |  |  |  |
| Platelets (x 10^9/L) | N | 60 | 60 | 0.244 | 63 | 63 | 0.013 | 0.371 | 150 - 450 |
|  | Mean | 260.67 | 253.27 |  | 285.30 | 277.92 |  |  |  |
|  | SE | 6.39 | 7.27 |  | 7.12 | 6.80 |  |  |  |
| WCC (x 10^9/L) | N | 60 | 60 | 0.864 | 63 | 63 | 0.267 | 0.549 | 4.0 - 11.0 |
|  | Mean | 6.41 | 6.38 |  | 6.25 | 6.06 |  |  |  |
|  | SE | 0.17 | 0.19 |  | 0.19 | 0.16 |  |  |  |
| Neutrophils (x 10^9/L) | N | 60 | 60 | 0.745 | 63 | 63 | 0.768 | 0.659 | 2.0 - 8.0 |
|  | Mean | 3.75 | 3.80 |  | 3.60 | 3.56 |  |  |  |
|  | SE | 0.13 | 0.16 |  | 0.15 | 0.12 |  |  |  |
| Lymphocytes (x 10^9/L) | N | 60 | 60 | 0.193 | 63 | 63 | 0.023 | 0.536 | 1.0 -4.0 |
|  | Mean | 2.01 | 1.95 |  | 2.00 | 1.89 |  |  |  |
|  | SE | 0.07 | 0.06 |  | 0.07 | 0.06 |  |  |  |
| Monocytes (x 10^9/L) | N | 60 | 60 | 0.496 | 63 | 63 | 0.129 | 0.461 | 0.2 -1.2 |
|  | Mean | 0.49 | 0.48 |  | 0.49 | 0.46 |  |  |  |
|  | SE | 0.02 | 0.02 |  | 0.02 | 0.02 |  |  |  |
| Eosinophils (x 10^9/L) | N | 60 | 60 | 0.616 | 63 | 63 | 0.423 | 0.349 | < 0.7 |
|  | Mean | 0.14 | 0.15 |  | 0.16 | 0.15 |  |  |  |
|  | SE | 0.01 | 0.01 |  | 0.01 | 0.01 |  |  |  |
| Basophils (x 10^9/L) | N | 60 | 60 | 0.811 | 63 | 63 | 0.199 | 0.300 | < 0.2 |
|  | Mean | 0.07 | 0.07 |  | 0.07 | 0.06 |  |  |  |
|  | SE | 0.01 | 0.01 |  | 0.01 | 0.01 |  |  |  |
| **Liver Function** | | | | | | | | | |
| Bilirubin (umol/L | N | 59 | 60 | 0.875 | 63 | 63 | 0.744 | 0.881 | < 16 |
|  | Mean | 8.98 | 8.97 |  | 8.52 | 8.40 |  |  |  |
|  | SE | 0.46 | 0.48 |  | 0.47 | 0.47 |  |  |  |
| ALP (U/L) | N | 59 | 60 | 0.914 | 63 | 63 | 0.114 | 0.187 | 30 - 110 |
|  | Mean | 71.29 | 70.62 |  | 71.37 | 68.65 |  |  |  |
|  | SE | 3.13 | 3.17 |  | 2.73 | 2.74 |  |  |  |
| GGT (U/L) | N | 59 | 60 | 0.653 | 63 | 63 | 0.498 | 0.747 | < 36 |
|  | Mean | 20.17 | 19.75 |  | 21.13 | 20.33 |  |  |  |
|  | SE | 1.78 | 1.58 |  | 2.81 | 3.46 |  |  |  |
| ALT (U/L) | N | 59 | 60 | 0.321 | 63 | 63 | 0.165 | 0.471 | < 36 |
|  | Mean | 22.76 | 21.85 |  | 21.24 | 19.29 |  |  |  |
|  | SE | 2.02 | 1.79 |  | 1.48 | 0.85 |  |  |  |
| AST (U/L) | N | 59 | 60 | 0.174 | 63 | 63 | 0.872 | 0.628 | < 36 |
|  | Mean | 23.03 | 22.13 |  | 22.79 | 22.62 |  |  |  |
|  | SE | 1.13 | 1.00 |  | 1.20 | 0.95 |  |  |  |
| Albumin (g/L) | N | 59 | 60 | 0.782 | 63 | 63 | 0.804 | 0.711 | 38 - 50 |
|  | Mean | 43.81 | 43.83 |  | 43.43 | 43.37 |  |  |  |
|  | SE | 0.30 | 0.28 |  | 0.32 | 0.25 |  |  |  |
| Total Protein (g/L) | N | 59 | 60 | 0.293 | 63 | 63 | 0.068 | 0.591 | 60 - 80 |
|  | Mean | 70.46 | 69.95 |  | 70.10 | 69.43 |  |  |  |
|  | SE | 0.56 | 0.51 |  | 0.47 | 0.44 |  |  |  |
| Globulin (g/L) | N | 59 | 60 | 0.091 | 63 | 63 | 0.047 | 0.856 | 22 - 38 |
|  | Mean | 26.64 | 26.05 |  | 26.67 | 26.06 |  |  |  |
|  | SE | 0.42 | 0.41 |  | 0.33 | 0.34 |  |  |  |
| **Renal Function** | | | | | | | | | |
| Sodium (mmol/L) | N | 60 | 60 | 0.328 | 63 | 62 | 0.243 | 0.434 | 135 - 145 |
|  | Mean | 139.25 | 137.60 |  | 139.62 | 139.31 |  |  |  |
|  | SE | 0.27 | 1.71 |  | 0.27 | 0.24 |  |  |  |
| Potassium (mmol/L) | N | 60 | 60 | 0.079 | 63 | 62 | 0.832 | 0.266 | 3.5 - 5.2 |
|  | Mean | 4.17 | 4.25 |  | 4.22 | 4.23 |  |  |  |
|  | SE | 0.04 | 0.04 |  | 0.04 | 0.05 |  |  |  |
| Chloride (mmol/L) | N | 60 | 60 | 1.000 | 63 | 62 | 0.361 | 0.565 | 95 - 110 |
|  | Mean | 102.47 | 102.47 |  | 102.71 | 102.52 |  |  |  |
|  | SE | 0.25 | 0.27 |  | 0.26 | 0.21 |  |  |  |
| Bicarbonate (mmol/L) | N | 60 | 60 | 0.869 | 63 | 62 | 0.320 | 0.427 | 22 - 32 |
|  | Mean | 27.28 | 27.33 |  | 27.95 | 27.71 |  |  |  |
|  | SE | 0.27 | 0.29 |  | 0.26 | 0.28 |  |  |  |
| Urea (mmol/L) | N | 60 | 60 | 0.455 | 63 | 62 | 0.532 | 0.859 | 3.0 - 8.0 |
|  | Mean | 5.53 | 5.39 |  | 5.86 | 5.79 |  |  |  |
|  | SE | 0.20 | 0.18 |  | 0.16 | 0.18 |  |  |  |
| Creatinine (umol/L) | N | 60 | 60 | 0.289 | 63 | 62 | 0.973 | 0.438 | 45 - 90 |
|  | Mean | 70.90 | 71.90 |  | 70.48 | 70.40 |  |  |  |
|  | SE | 1.30 | 1.50 |  | 1.06 | 1.09 |  |  |  |
| eGFR (count) (mL/min/1.73m^2) | N | 60 | 60 | NA | 63 | 62 | NA | NA | > 90 |
|  | ≥ 90 | 22 | 21 |  | 28 | 27 |  |  |  |
|  | < 90 | 38 | 39 |  | 35 | 35 |  |  |  |

^a^P-values are generated from a paired-samples T-test.

^b^P-values are generated from an independent-samples T-test (change from day 0 to day 28).
